# Marine Biodiversity and Biogeography – Regional Comparisons of Global Issues, an Introduction

**DOI:** 10.1371/journal.pone.0011871

**Published:** 2010-08-02

**Authors:** Ron O'Dor, Patricia Miloslavich, Kristen Yarincik

**Affiliations:** 1 Census of Marine Life Secretariat, Consortium for Ocean Leadership, Washington, D. C., United States of America; 2 Departamento de Estudios Ambientales, Universidad Simón Bolívar, Caracas, Miranda, Venezuela; University of Hull, United Kingdom

The Census of Marine Life grew out of a series of global concept meetings, following a 1995 U.S. National Research Council report indicating that no nation in the world could list the species that live in its offshore exclusive economic zone, as required under the Convention on Biological Diversity. A series of “Known, Unknown, Unknowable” meetings convened in every ocean region enabled the Census Scientific Steering Committee to compile information and enlist people from around the world to form a network of National and Regional Implementation Committees in support of a global effort to resolve this knowledge void. This collection represents the assembled results of their activities. As these data are incorporated into the Census' Ocean Biogeographic Information System and made available through its new home at the UN's Intergovernmental Oceanographic Commission, they will be the best records of most countries' marine biodiversity. The people, technologies, and associations of the Census will play a key role the UN General Assembly's future Regular Marine Assessments. This collection is a major legacy of the Census' US$650 million “Decade of Discovery” to be released in London in October 2010.

## Introduction

By the mid-1990s, it was already clear that the oceans, routinely treated as limitless sources and sinks for human consumption and waste, were changing in response to intense fishing, pollution, and climate change [1]. Fred Grassle, as a member of the U.S. National Research Council committee that wrote a report advising a scientific approach to studying marine biodiversity, initiated discussions with Jesse Ausubel, a program officer for the Alfred P. Sloan Foundation, recognizing that science had sampled less than 0.1% of the volume of the oceans. The outcome of their discussions was a series of concept meetings focusing on the question of whether it was possible to document what lives in the world oceans, so that the changes could be monitored and understood [2]. Taking place from 1997 to 1999, the meetings resulted in a recommendation for a comprehensive international research program called the Census of Marine Life. The purpose of the Census was to assess and explain the diversity, distribution, and abundance of marine organisms throughout the world's oceans [3].

The oceans occupy over 70% of the earth's surface area and over 90% of its biosphere's volume. Thus, documenting the life that exists in this part of the planet is a huge challenge. The 300 scientists who participated in the concept meetings, however, agreed that new technologies becoming available at the turn of the millennium made it feasible to ask and answer these questions. Although the precision of the Census was not predetermined, and costs were estimated to be in the billion-dollar range, major advances were possible on a schedule that could contribute usefully to the understanding required to manage an environment under increasing pressure. In view of the cost of mounting a global-scale research program, the Census focused initially on assembling existing information. In addition to ongoing international and governmental research and monitoring programs, industries such as transportation, fishing, oil exploration, and mining sample the ocean continuously in a variety of ways. Thus, cooperation among scientists and stakeholders, along with the use of computer and Internet technology, were crucial to bringing global data and expertise together to assess life in the oceans.

The scope of the Census program quickly made it clear that its scale would require a new sort of global collaboration [2], [4] to achieve its goals. This necessity was the origin of the National and Regional Implementation Committees (NRICs), the main source of the information assembled in this collection. Because of the complex evolution of the program and the NRICs, some context is essential to understand the origins of the information.

## Background

In 1999, a group of senior marine scientists from around the world formed the Scientific Steering Committee (SSC) for the Census. In a meeting under the aegis of the UN Intergovernmental Oceanographic Commission (IOC) in Paris in 2000, this group realized its first goal for the Census – the development of a data management system to assemble existing information and make it accessible to scientists around the world. The Ocean Biogeographic Information System – OBIS (www.iobis.org) – now a global-scale, Internet-based system of interoperable databases, was designed to provide a new baseline of knowledge of marine systems. By 2010, it was to provide not only access to maps of the distribution and abundance of living organisms, but also links to the chemical and physical characteristics of the environment in which they live.

**Figure 1:**
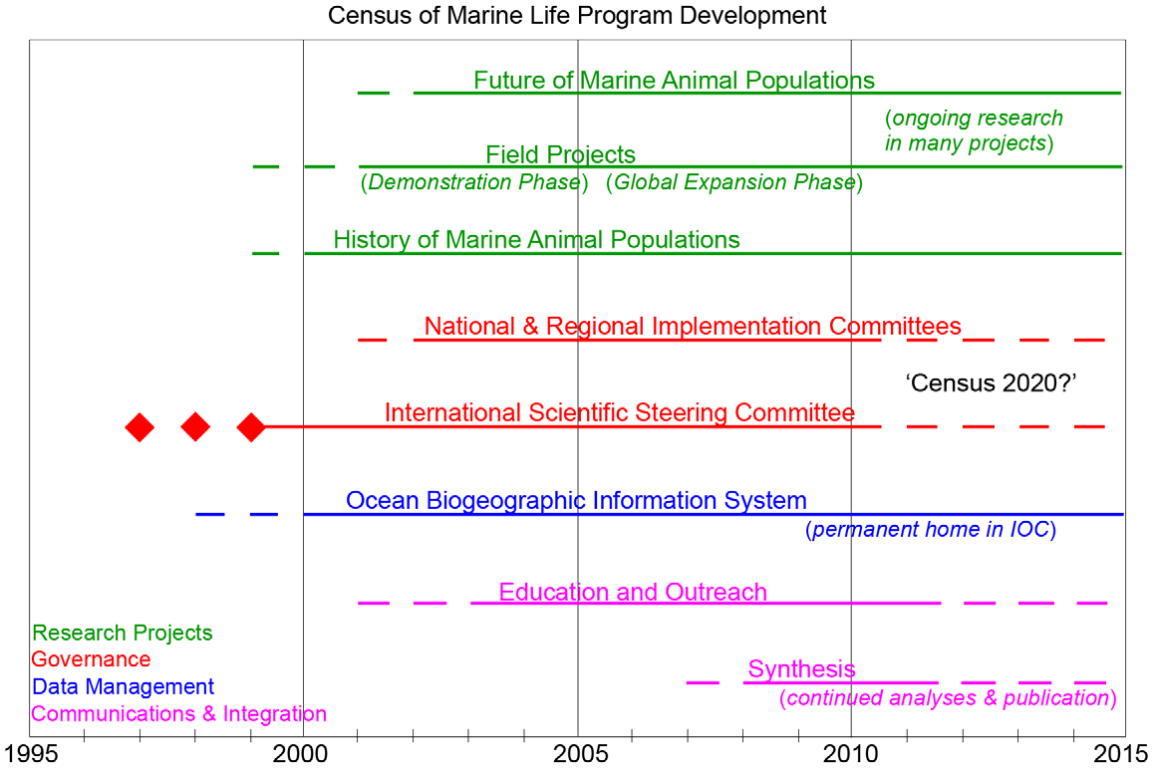
Historical development of the Census of Marine Life and projected continuation of its various components. The Census timeline began with the concept meetings in 1997 and the implementation of the program and its components followed as represented in this timeline. While the Census program will conclude at the end of 2010, many of its components will continue independently beyond 2010.

**Figure 2:**
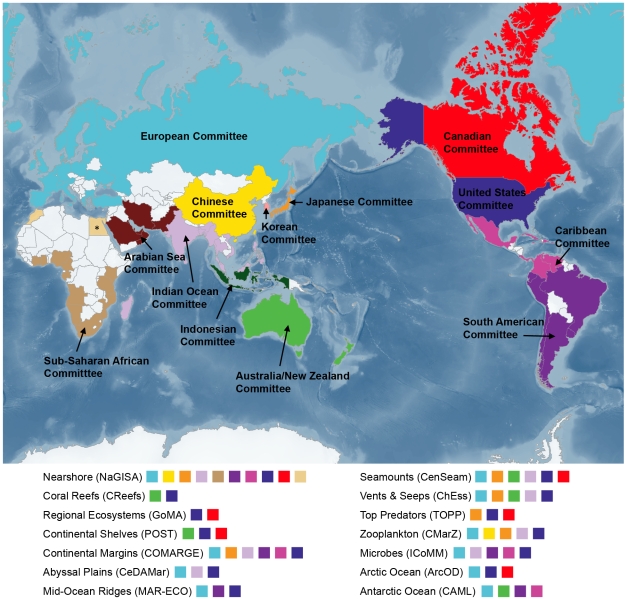
Countries encompassed by the NRICs from 2001 to 2010 and their field project interactions. This map, with each NRIC indicated in a different color, shows the countries encompassed by each NRIC. Below, the same colors indicate which NRICs played major roles in globalization of Census field projects. The success of NaGISA as an “ambassador” project is evident as almost all NRICs participated. All NRICs had strong involvement in the Ocean Biogeographic Information System, but some (Korea, Indonesia, Arabian Sea) focused solely on data assembly. (* Morocco and Egypt participated in the Census outside of NRIC auspices.) See www.coml.org for further details on the projects and acronyms.

The next step was the development of a series of projects to collect new information on life in the oceans (Figure 1). These projects comprise elements dealing separately with the past, present, and future. To address the *past*, the Census initiated the History of Marine Animal Populations (HMAP) to assemble and analyze historical data from around the world on the distribution and abundance of marine organisms before the era of modern fisheries management. Historians, anthropologists, ecologists, and biologists teamed together to glean historical data from sources as diverse as old whaling logs and seafood menus. This information could then be combined with current data in mathematical ecosystem models to predict the future state of marine communities. This rescued history has created a new vision of ocean life as it existed before major human impacts and provided a historical context for new information collected by the NRICs. The Census addressed the *present* through a series of field projects (Figures 1 and 2), fostered by the SSC and designed to demonstrate new quantitative approaches for sampling a full spectrum of life forms in the major ocean habitats, but requiring local interest and funding to expand (Table 1 and Figure 2). The field projects eventually grew to 14, each with international collaboration and standardized approaches, mostly global in scope. The field projects, HMAP, and OBIS were knit together in the *future* component. The Future of Marine Animal Populations (FMAP) is a modeling project that used past and present data to project possible futures, as well as to model regional data to predict patterns at larger scales.

**Table 1:** Summary of regional activities contributing to the globalization of the Census of Marine Life.

NRICs	Start	Project Headquarters	Affiliates	Major Synthetic Publications	OBIS Nodes
Canada	2/02	POST, FMAP	OTN, CHONe	Three Oceans of Biodiversity [13]	1
Japan	3/02	NaGISA			1
EU	9/02	HMAP, MAR-ECO, CeDAMar, ChEss, COMARGE	EUTOPIA, EMBED		1
S. America	10/02			First South American Workshop on Marine Biodiversity [14]	3
USA	12/02	OBIS, GoMA, TOPP, CMarZ, ArcOD, ICoMM	GoMx	Managing for ocean biodiversity to sustain marine ecosystem services [15]	1
Australia/NZ	1/03	CenSeam, CAML, CReefs	GBRSB		2
SS Africa	9/03			Marine Biodiversity in Sub-Saharan Africa: The Known and Unknown [16]	1
Indian Ocean	12/03			Coastal and Marine Biodiversity of the Indian Ocean [17]	1
China	4/04			Checklist of Marine Biota of China Seas [18]	1
Caribbean	6/04			Caribbean Marine Biodiversity: The Known and Unknown [19]	
Indonesia	7/07			Marine Biodiversity Review of the Arafura and Timor Seas [20]	
Arabian Sea	10/07				
Korea	10/07				1
**Regional Projects**					
ArcOD		Arctic Council affiliate		Proceedings of the Arctic Biodiversity Workshop [21]	
CAML		Scientific Committee on Antarctic Research affiliate		First insights into the biodiversity and biogeography of the Southern Ocean deep sea [22]	1

Pacific Ocean Shelf Tracking Project (POST), Future of Marine Animal Populations (FMAP), Natural Geography in Shore Areas (NaGISA), History of Marine Animal Populations (HMAP), Patterns and Processes of Ecosystems in the Northern Mid-Atlantic (MAR-ECO), Census of Diversity of Abyssal Marine Life (CeDAMar), Biogeography of Deep-Water Chemosynthetic Ecosystems (ChEss), Continental Margin Ecosystems on a Worldwide Scale (COMARGE), Ocean Biogeographic Information System (OBIS), Gulf of Maine Area (GoMA), Tagging of Pacific Predators (TOPP), Census of Marine Zooplankton (CMarZ), Arctic Ocean Diversity (ArcOD), International Census of Marine Microbes (ICoMM), Global Census of Marine Life on Seamounts (CenSeam), Census of Antarctic Marine Life (CAML), Census of Coral Reefs (CReefs), Ocean Tracking Network (OTN), Canadian Healthy Oceans Network (CHONe), European Tracking of Predators in the Atlantic (EUTOPIA), Environmental Modulation of Biodiversity Ecosystem Dynamics (EMBED), Gulf of Mexico Biodiversity (GoMx), Great Barrier Reef Seabed Biodiversity (GBRSB).

Building on an extensive series of topical and geographical reviews of the known, unknown, and unknowable (KUU, Table 2) aspects of marine biodiversity, a Research Plan [5], [6] defined the approaches the Census would develop before its decadal report in 2010. Drawing on experience in a range of disciplines and political systems, the SSC oversaw the research program and immediately recognized the need for a much broader community with regional and specialized technical expertise and helped to bring it together. Achieving global coverage for the Census projects depended on the efforts of the NRICs, which were just starting to form as well. The nearshore project, NaGISA, one of the earliest projects introduced, was an ideal “ambassador” project for this purpose because its low-budget, low-tech approach made it easy to implement in new regions. Its interaction with nearly all of the NRICs (Figure 2) suggests that it succeeded.

**Table 2:** International and regional organizations involved in “known, unknown, and unknowable” (KUU) workshops; countries and academic institutions engaged.

NRIC/Establishment Date	Countries engaged	Institutions	Intergovernmental organizations/programs
Arabian Sea/Gulf of Oman (10/07)	9	18	IOC^1^
Australia (1/03)	1	8	
Canada (2/02)	3	26	WWF^2^
Caribbean (6/04)	18	31	IOC, Caribbean Coastal Marine Productivity Program, Association of Marine Laboratories of the Caribbean, Ocean Tracking Network, The Nature Conservancy, Conservation International, Chevron, ConocoPhillips, Petróleos de Venezuela, Caribbean Large Marine Ecosystem
China (*) (6/04)	1	9	National Natural Science Foundation of China
EuroCoML (9/02)	11	18	IABO/IAPSO^3^, Niarchos Foundation, International Council for the Exploration of the Sea
Indian Ocean (12/03)	16	21	IOC, POGO^4^
Indonesia (7/07)	2	5	Global Environmental Facility Arafura and Timor Seas Ecosystem Action
Japan (*) (3/02)	2	11	IOC, POGO, NIPPON Foundation, Diversitas
Korea (10/07)	1	1	
South America (10/02)	15	26	POGO
Sub-Saharan Africa (9/03)	17	25	WWF, Global Invasive Species Programme, Marine Species Database for Eastern Africa, Ocean Data and Information Network for Africa, Seaweed Africa Database, Seawaste Network
United States (12/02)	1	35	
**TOTAL**	**65**	**236**	**22**

(*)Before these national committees were established, there was an initial KUU workshop in the South East Asia region with participation from 14 countries and 31 institutions. The intergovernmental organization involved in this event was the IOC^1^.

1 Intergovernmental Oceanographic Commission of UNESCO and its regional affiliates.

2 World Wide Fund for Nature and its global affiliates.

3 International Association for Biological Oceanography and International Association for the Physical Sciences of the Ocean.

4 Partnership for the Observation of Global Oceans.

Further support for the NRICs came as the Census' Secretariat shifted from project development to program development, emphasizing coordination among national and regional committees, facilitating activities related to Census projects, and linking them to local and regional marine biodiversity initiatives. The Secretariat worked to coordinate regional funding and build partnerships that highlighted its role as a global aggregator of data and information, such as by serving as a Biology Editor for the UN Atlas of the Oceans. Agencies such as the UN and World Bank through the Global Environment Facility (GEF) and Large Marine Ecosystem (LME) programs were interested in proposals from cooperating groups of countries but the timelines for developing these applications were generally much longer than those of the Census, so the results were typically improved collaborations with existing programs, rather than new ones. To ensure global interest and participation, the Secretariat and the Sloan Foundation worked to distribute management teams for projects around the globe to encourage countries, regions, and scientists to take the lead in field projects for which they had special interests or capacity (Table 1).

The individual NRICs grew out of the geographically focused KUU meetings (Tables 1 & 2). Building on its links to the IOC, the SSC sponsored its first regional meeting in October 2001 in Phuket, Thailand. Although the Western Pacific region was linked within the IOC, national requirements for biodiversity information under the Convention on Biological Diversity (CBD) ultimately led to the formation of several NRICs as an outcome of this meeting. Because the NRICs were expected to take responsibility for Census legacy projects in their waters and regions, their evolution was a complex process, mixing geography, funding, and politics. The SSC began with the view that NRICs should naturally form around shared bodies of water that would generate common research goals, but the practicalities of funding and the relatively complex legal framework for sharing such waters led the NRICs in different directions.

The IOC Western Pacific region ultimately became a series national committees in Japan, China, Korea, and Indonesia. Surprisingly, Europe became the first regional committee, recognizing that the common theme of ocean research could be a strong attractor of European Union funding, even though it reached all the way from the Atlantic to the Arctic to the Pacific. Most other committees followed suit this way, choosing either national or regional organization based on political necessity, funding structures, or the benefits of cooperation. Ocean foci did develop in the Indian Ocean, Caribbean, and the Arabian Sea. Census studies in the Arctic and Southern oceans appeared likely candidates for a water focus, but territorial disputes in the Arctic seem to be mirrored in this collection in that the U.S., Canada, and Europe each have written about it. The Arctic Ocean field project did, however, produce a unified KUU report early on, similar to those of the NRICs (Table 1), as well as a final synthesis of new information [7]. In the Southern Ocean, the Census' Antarctic field project, which operates under the aegis of the Scientific Committee for Antarctic Research formed by the Antarctic Treaty organizations, is the unifying force and has reported in this collection. The relationships among the 13 NRICs are complex, but, as Figure 2 shows, there is nearly comprehensive coverage of the nations of the world with ocean interests and capacity.

The assembly of data on regional species done in preparation for the KUU workshops on the NRIC regions led some NRICs to take responsibility for regional OBIS nodes that compiled and redistributed biodiversity records in the nations or regions. The regional nodes make it possible to summarize and specialize OBIS data to better serve local requirements and provide a level of control over content and quality. Many of the regional nodes were directly supported by local governments, which helped transform OBIS from an independent database to its new role as a permanent biodiversity resource.

Many of the Census field projects used the efforts of the NRICs to identify key research targets in the regions and to enhance local support for marine research. This support included not only providing funds needed to build bridges between Census projects and government initiatives such as LME programs, but also simply using local influence to obtain access for researchers to exclusive economic zones or to acquire permits to use and export biological materials. This latter form of support has been particularly valuable for Census projects that have made extensive use of barcodes and other DNA technologies. The Caribbean, South American, and Indian Ocean Committees have been of great assistance to the Census project on marine microbes, for example, and the powerful alliance of the Census and the Barcode of Life projects has helped in building capacity, in moving samples between countries, and in processing samples in countries where facilities had to be built specifically for that purpose.

Another major role of NRICs has been the “affiliation” of projects with funding horizons beyond 2010. Among these are regional projects like The Gulf of Mexico—Past, Present, and Future through the U.S. and the Great Barrier Reef Seabed Biodiversity Project through Australia. NRICs around the world were instrumental in the formation of the Ocean Tracking Network, a Canadian affiliated project that links over a dozen countries distributed over 14 ocean regions, using the tools demonstrated by the Census' Top Predators and Continental Shelves tracking projects to follow movements and interactions of marine animals, as well as monitoring the conditions they experience. The Ocean Tracking Network is a Global Ocean Observing System project, another IOC mechanism for regular assessment of the oceans. Census outputs are becoming a permanent part of the IOC and of observing systems.

## Discussion

In 2007, the Census began discussing the best strategy to synthesize its results so that they would provide a useful and comprehensive view of the program's discoveries to a wide audience including scientists, educators, policy makers, governmental and nongovernmental parties, and the general public. The Census surveyed these various audiences to determine what scientific products would be most useful for them and in which formats. The Census Synthesis Group, established to coordinate the production of the end products, took this survey information and began developing, among other things, the collection of national and regional papers introduced here: “Marine Biodiversity and Biogeography – Regional Comparisons of Global Issues.”

The genesis of this collection came in May 2008 through a meeting of all the leaders of the Census NRICs, along with the Census' mapping and visualization experts and OBIS. The participants agreed on the need for a worldwide compilation of marine biodiversity information from both national and regional perspectives to update the information presented in the earlier KUU workshops and include the Census field projects' discoveries in these regions. As mentioned earlier, a total of 13 of these committees compose the Census. However, to have a wider review, it was agreed that the collection would greatly benefit from adding the Southern Ocean (Antarctic waters) and having Australia and New Zealand report separately. At a follow-up meeting in February 2009, the content of this collection was further discussed and the guidelines and templates were finalized among the NRIC coordinators, authors, and *PLoS ONE* editors.

An important aspect of this collection is that it constitutes an assessment of the knowledge of global marine biodiversity with a common approach and the added value of consistent quality of information. Following the structure of a research article, these papers have an introduction in which the regions and offshore boundaries are defined and some regional statistics are provided (sea area, length of coastline, numbers of countries, etc.), along with maps showing depth contours, habitat types, and other features. This section also includes basic information on physical, geological, chemical, and biological characteristics of the region (current and temperature regimes, depth distributions, types and areas of various ecosystems, etc.), a brief history of research and species discovery in the region, and some insight into the role of Census activities in promoting and synthesizing this information. In developing the papers, the NRICs compiled and analyzed information on national or regional marine biodiversity (species lists), research capacity (approximate numbers and ages of marine laboratories), and taxonomic capacity (number of systematists, major collections).

When possible, each NRIC indicates sampling distribution and intensity and discusses how these attributes are distributed by geographic region, by method, by date, by ecosystem, and by depth. Each article includes a summary table presenting these results as the national or regional species richness by taxonomic group; supporting information tabulates these data for all phyla and includes data on endemic and introduced species. In many of the articles, these results are presented in a spatially explicit or graphical form such as species richness mapped as hot spots, species per 100 km of coast, or species by habitat or by ecosystem. Finally, the authors of each paper discuss their analysis in the context of what is known, unknown, and unknowable about marine biodiversity in their study area. They do this by explaining, for example, whether the data are realistic or whether it is clear that certain regions or taxa are underrepresented. The biases introduced by available taxonomic expertise, both historical and present, available capacity, types of sample taken, and so forth, are also discussed. The papers comment briefly on value, use, and impacts of marine biodiversity in the countries or regions, on the major threats to biodiversity, on conservation areas, and on potential and priorities for future discovery and research. The final paper of the collection [8] provides an integrated analysis of the findings of all these national and regional papers to provide a global perspective on what is known, what are the major gaps in information about marine biodiversity, and what are the major threats to its sustainability.

The benefit of this collection, at both the national and the regional level, will be far reaching. There is intrinsic value in pulling together the hundreds of references and research reports that went into these papers, which will now be available globally through a single source. Most of the information in these papers was previously distributed widely and difficult to access even to experts, let alone policy makers. To compile it, a team of more than 100 scientists, led by taxonomists, was necessary.

This collection of regional biodiversity surveys provides a valuable contribution to future ocean governance. The UN General Assembly has called for a regular marine assessment in the future, but in the recently published Marine Assessment of Assessments [9], the first step toward this goal, biodiversity was comparatively weakly represented despite participation of some NRIC members. Further, the Assessment of Assessments points out, “There is no commonly agreed regional division of the world's oceans; several divisions exist for different purposes, often not covering the whole ocean area” [9; see Annex 1]. What would make better sense than divisions based the biogeography of the creatures that live in the ocean, rather than on the creatures that live on land [10]? We hope that these national and regional papers, along with the papers of the field projects [11], can serve as a more comprehensive baseline for future assessment.

The SSC anticipates that the Census community will maintain its momentum, develop new research goals, and build toward another 10 years of research. The NRICs are well placed to stabilize Census legacies and focus on regional issues and societal benefits. Future assessments could certainly benefit from Census expertise and its mechanisms for conducting good science amid political pressures [4]. This collection will strengthen both the basis for regular assessment and the mechanisms for doing it. Thanks to NRICs urging national representatives' support at the 2009 IOC Assembly, OBIS was assured a continuing existence as a contributor to the International Oceanographic Data and Information Exchange (IODE) system. Under the Convention on the Law of the Sea, the UN is the only government the ocean has, and the Census has certainly demonstrated the crucial need for mechanisms to ensure that good science is incorporated into its management (Table 2).

Overall, the Census community has been remarkably successful in this huge undertaking. The Census has been widely recognized as providing real science in support of ocean biodiversity policies that were previously based largely on politics [12]. However, the job is not complete. There are still vast volumes of ocean that are virtually unknown and clear evidence that even what we know now will be changing rapidly with climate over the next decades. The NRICs have developed at different times within the life of the program, often with different goals and tools. Some were primarily interested in raising funds to support projects, while others sought to bring the Census vision into their countries or regions. Whatever the sources, the Census managed to spend a high proportion of its budget on science, and while it is possible to conduct politics in the absence of science, the knowledge provided by science is fundamental to good politics. Good politics supports science for the benefit of society, and the relationship should be one of mutual dependence. The Census community seems to have established a reputation for providing good information on a global scale, and the NRICs provide credibility and a collaborative spirit. In return for the global support that has made the Census possible, the Census community has a collective responsibility to continue to get the best answers we can and to communicate them widely. We hope that our science will continue to benefit many societies, and the biodiversity that we share will keep us working together.
